# Population history of deep-sea vent and seep *Provanna* snails (Mollusca: Abyssochrysoidea) in the northwestern Pacific

**DOI:** 10.7717/peerj.5673

**Published:** 2018-09-26

**Authors:** Tomomi Ogura, Hiromi Kayama Watanabe, Chong Chen, Takenori Sasaki, Shigeaki Kojima, Jun-ichiro Ishibashi, Katsunori Fujikura

**Affiliations:** 1 Graduate School of Marine Science and Technology, Tokyo University of Marine Science and Technology, Tokyo, Japan; 2 Japan Agency for Marine-Earth Science and Technology (JAMSTEC), Yokosuka, Kanagawa, Japan; 3 The University Museum, The University of Tokyo, Tokyo, Japan; 4 Atmosphere and Ocean Research Institute, The University of Tokyo, Kashiwa, Chiba, Japan; 5 Department of Earth and Planetary Sciences, Faculty of Science, Kyushu University, Fukuoka, Japan

**Keywords:** Chemosynthetic community, DNA barcoding, Population expansion, Okinawa Trough

## Abstract

**Background:**

Gastropods of the genus *Provanna* are abundant and widely distributed in deep-sea chemosynthetic environments with seven extant species described in the northwestern Pacific.

**Methods:**

We investigated the population history and connectivity of five *Provanna* species in the northwestern Pacific through population genetic analyses using partial sequences of the cytochrome *c* oxidase subunit I gene.

**Results:**

We found that *P. subglabra*, the most abundant and genetically diverse species, is genetically segregated by depth. Among the five species, the three comparatively shallower species (*P. lucida, P. kuroshimensis, P. glabra*) had a more constant demographic history compared to the deeper species (*P. subglabra*, *P.  clathrata*).

**Discussion:**

Environmental differences, especially depth, appears to have a role in the segregation of *Provanna* snails. The population of *P. clathrata* in the Irabu Knoll appears to have expanded after *P. subglabra* population. The remaining three species, *P. lucida*, *P. kuroshimensis*, and *P. glabra*, are only known from a single site each, all of which were shallower than 1,000 m. These data indicate that *Provanna* gastropods are vertically segregated, and that their population characteristics likely depend on hydrothermal activities.

## Introduction

A true understanding of biodiversity is not just about counting the number of species, but must also encompass comprehensive understanding of evolutionary processes and natural history in a heterogeneous biosphere (Grosberg, Vermeij & Wainwright, 2012). The knowledge of evolutionary processes in the deep-sea, despite it making up about 95% of all habitable space on Earth, is limited due to its inaccessibility. Deep-sea hydrothermal vents and hydrocarbon seep areas are populated by many species endemic to these deep habitats, and provide opportunities to investigate evolutionary and ecological processes, such as succession and the invasion of new habitats. About half of known hydrothermal vents are located on mid-ocean ridges, while the other half are located in arc-backarc systems (Beaulieu et al., 2013). The latter systems are younger, for example, vents in the Mariana Trough and Manus Basin appeared only six and four million years ago, respectively (Ishibashi & Urabe, 1995). Unlike vents, hydrocarbon seeps are not confined to spreading centres, and are found along passive and active margins around the globe. Faunal compositions of vent and seep fauna tend to be different, and fauna in back-arc hydrothermal vents are mostly separated among basins (Desbruyères, Hashimoto & Fabri, 2006). To understand evolutionary processes in the deep sea, it is critical to understand how taxa invade and colonize such ecosystems.

The northwestern Pacific is an excellent model system for examining such evolutionary processes, since there are back-arc vents (e.g., Okinawa Trough, Manus Basin) and hydrocarbon seeps (e.g., Sagami Bay, Nankai Trough) in close proximity. The Okinawa Trough is a back-arc basin with more than 10 known hydrothermal vent fields, and more continue to be discovered (Ishibashi et al., 2015; Nakamura et al., 2015; Chen et al., 2017; Miyazaki et al., 2017). As these vents appeared only two million years ago at the southern end of the back-arc basin, the historical evidence of animals invading these habitats can still be recovered genetically, along with ongoing evolutionary and ecological processes. Gastropod snails of the superfamily Abyssochrysoidea radiated about 50–158 million years ago, and today they are common inhabitants of deep-sea chemosynthetic environments including hydrothermal vent fields, hydrocarbon seep sites, and organic falls (Johnson et al., 2010). The genus *Provanna,* particularly, is a representative group in the northwestern Pacific (Fujikura, Okutani & Maruyama, 2008; Johnson et al., 2010). Recently, a combined effort of morphological and genetic approaches revealed that the *Provanna* species dominant in the Sagami Bay seep area is distinct from those (at least three species) inhabiting the Okinawa Troughvents. Furthermore, another species was discovered from the Kuroshima Knoll seep area in the Ryukyu Trench, on the eastern side of the Ryukyu Arc (Sasaki et al., 2016). However, ecological differences amongst species have not been available for these species, as *Provanna* species are difficult to differentiate in video imaging due to morphological similarities and their small sizes (as noted by Nakajima et al., 2015).

For such animals inhabiting deep-sea ecosystems that can only be observed in sporadic time points, population genetics provide valuable insights to inferring historical parametres such as dispersal, past population sizes, and relationships among populations, as demonstrated by Rogers & Herpending (1992). Indeed, population genetics studies have revealed many dispersal barriers for deep-sea hydrothermal vent faunas since the 1990s, including depth, oceanic currents, and lateral offsets in mid-oceanic ridges (reviewed in Vrijenhoek, 2010). Most of these dispersal barriers do not apply to arc-backarc and subduction systems which have different geological features and deep currents, however, and therefore dispersal barriers for deep-sea taxa remain poorly understood for the western Pacific where such systems dominate (Watanabe et al., 2010). Population genetic analyses of animals inhabiting the western Pacific will provide new insights for evolutionary and ecological processes shaping the deep-sea ecosystems as we know today across time, and help to improve dispersal models (Mitarai et al., 2016).

Accordingly, we conducted population genetic analyses of five *Provanna* species inhabiting the northwestern Pacific vents and seeps to understand their population history. One species, *P. glabra*, dominates the hydrocarbon seeps in Sagami Bay and has been shown to be phylogenetically very close to *P. laevis* in Monterey Bay, California (Sasaki et al., 2016). Three species, including *P*. *subglabra, P*. *clathrata*, and *P*. *lucida*, inhabit hydrothermal vents in the Okinawa Trough, with *P. subglabra* being the most abundant and wide-spread. A further species, *P. kuroshimensis,* is limited to the Kuroshima Knoll seep site in the Ryukyu Trench (Sasaki et al., 2016). For each species we investigated genetic diversities, which accumulates after colonization. Furthermore, we discuss the colonization scenario of the five *Provanna* species inferred from their population genetics data, and the potential factors contributing to their population history.

## Materials and Methods

### Sampling

**Figure 1:**
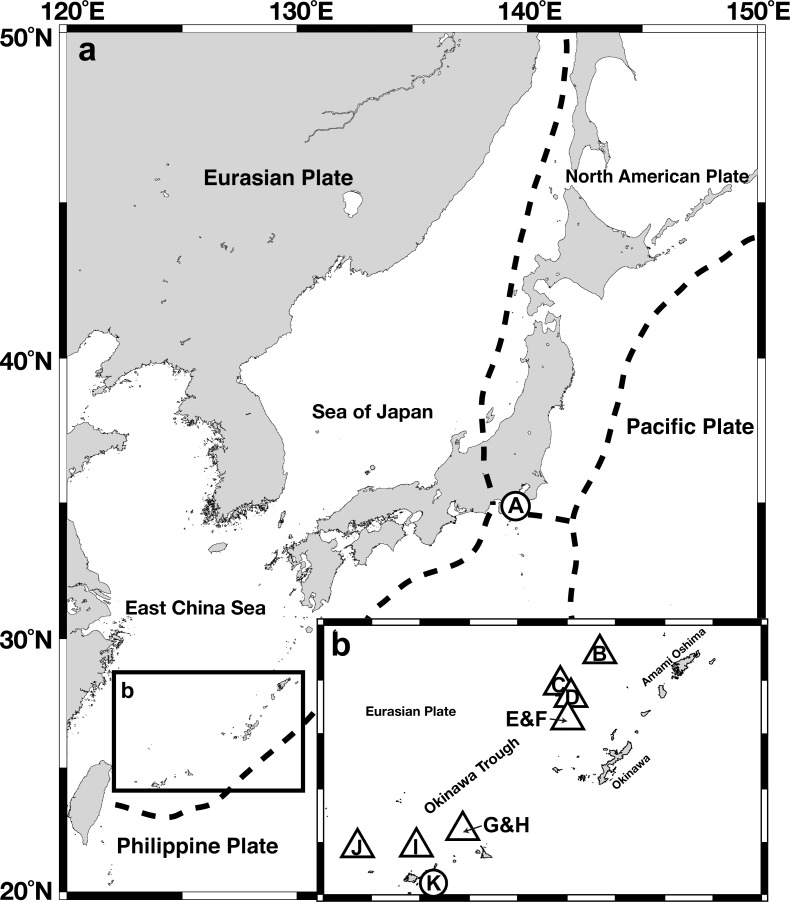
Gross map (A) and magnified map (B) of sampling locations in the Nansei-shoto area. Circles, methane seep sites; triangles, hydrothermal fields. IDs in the capitalized letters corresponds to those in Table 1.

A total of 204 *Provanna* gastropods were collected from 10 chemosynthetic sites in Sagami Bay, the Okinawa Trough, and the Ryukyu Trench (Fig. 1, Table 1), on-board the Japan Agency for Marine-Earth Science and Technology (JAMSTEC) ship R/V *Natsushima*. *Provanna glabra* was collected from the Off Hastushima seep site, Sagami Bay, by the remotely operated vehicle (ROV) *Hyper-Dolphin* during the NT11-01 cruise (January 2011). Specimens from hydrothermal vent fields in the Okinawa Trough were collected by the same submersible during the cruise NT11-20 conducted in October 2011. *Provanna kuroshimensis* was collected from the Kuroshima Knoll seep site, Ryukyu Trench, during the NT02-07 and NT02-08 cruises in May 2002 by ROV *Dolphin 3K* and the human-occupied vehicle (HOV) *Shinkai 2000*, respectively. Upon recovery on-board the ship, all specimens were fixed and preserved in 99.5% ethanol or frozen in −20 °C for DNA extraction.

**Table 1:** Sampling locations. IDs correspond to sampling sites in Fig. 1.

ID	Site	Type	Latitude	Longitude	Depth (m)
**Sagami Bay**					
A	Off Hatsushima	Seep	35°00′N	139°14′E	1,172
**Nansei-shoto area**					
B	Minami Ensei Knoll	Vent	28°24′N	127°38′E	701
C	Iheya North	Vent	27°48′N	126°54′E	982
D	Iheya Ridge	Vent	27°33′N	126°58′E	1,399
E	Jade site, Izena Hole	Vent	27°16′N	127°04′E	1,309
F	Hakurei site, Izena Hole	Vent	27°15′N	127°04′E	1,617
G–H	Irabu Knoll	Vent	25°14′N	124°52′E	1,646
I	Hatoma Knoll	Vent	24°52′N	123°51′E	1,473
J	Dai-yon Yonaguni Knoll	Vent	24°51′N	122°42′E	1,387
K	Kuroshima Knoll	Seep	24°08′N	124°12′E	644

### DNA extraction and sequencing

Genomic DNA was extracted from the foot muscle *Provanna* specimens using the DNeasy Tissue Extraction Kit (QIAGEN, Valencia, CA, USA), and 1 µL of the extraction was purified using GeneReleaser (BioVenture, San Carlos, CA, USA) following the manufacturer’s protocol. Fragments of the mitochondrial cytochrome *c* oxidase subunit I (COI) gene were amplified by polymerase chain reaction from 204 specimens, using the univcrsal primers LCO1490 and HCO2198 (Folmer et al., 1994) and the Pg501L and Pg1253R primer pair designed for *Provanna* (Sasaki et al., 2016). Reaction volumes consisted of 1 µL template DNA, 13.35 µL deionized sterilized water, 2 µL 10 × PCR buffer, 1.5 µL 2.5 mM dNTP, 1 µL of each primer, and 0.15 µL of 5 U/µL Ex *Taq* DNA polymerase (TaKaRa, Shiga, Japan), for a total of 20 µL. Targets were amplified by initial denaturation at 94 °C for 120 s, 30 cycles of denaturation at 94 °C for 30 s, annealing at 45 °C for 30 s, and extension at 72 °C for 30 s, followed by final extension at 72 °C for 40 s. PCR products were purified using ExoSAP-IT (United States Biochemical), and sequenced using the BigDye^®^ Terminator Cycle Sequencing Kit Version 3.1 (Applied Biosystems, Foster City, CA, USA). Sequencing reactions contained 1 µL of purified PCR products, 7.55 µL deionized sterilized water, 0.7 µL 5 × BigDye Sequencing Buffer, 0.25 µL of each primer, and 0.5 µL BigDye^®^, for a total of 10 µL. Reactions were initially denatured at 96 °C for 60 s, and then cycled 25 times at (96 °C for 10 s, 50 °C for 50 s, 60 °C for 60 s). The resulting products were purified using the BigDye XTerminator^®^ Kit (Applied Biosystems, Thermo Fischer) or a Gel Filtration Cartridge (Edge BioSystems, Gaithersburg, MD, USA), and sequenced using an ABI 3130 automated DNA sequencer (Applied Biosystems, Foster City, CA, USA). Sequences obtained were aligned, checked by eye, assembled, and translated into amino acids to confirm the absence of stop codons in Geneious v9 (http://www.geneious.com, Kearse et al., 2012). The resulting COI sequences were registered to DDBJ, EMBL, and GenBank under Accession Numbers AB810040 to AB810216 (Table S1).

### Population genetic analyses

Parsimonious haplotype networks were reconstructed for each species from mitochondrial COI fragments (1,044 bp) using the program TCS ver. 2.01 (Clement, Posada & Crandall, 2000) with the connection probability set to 95% and sequences differing only by ambiguous characters treated as the same haplotype. Population history was inferred from the same mitochondrial COI fragmentsfrom about 20 specimens per population (Table 1, Fig. 1) in the program ARLEQUIN ver. 3.5.1.2 (Schneider, Roessli & Excoffier, 2000). Nucleotide and haplotype diversity were estimated, and parametres for the available populations were compared by pairwise Φ_*ST*_ comparison of haploid genes and exact tests (Raymond & Rousset, 1995), as well as AMOVA assuming two scenarios: (1) latitudinal subdivision into northern (Iheya North, Iheya Ridge, JADE and Hakurei sites in the Izena Hole) and southern (Irabu, Hatoma and Dai-yon Yonaguni) populations, or (2) depth subdivision into shallower (Iheya North, Iheya Ridge, and JADE site in Izena Hole) and deeper (Hakurei site in Izena Hole, Irabu, Hatoma and Dai-yon Yonaguni Knoll) populations. Genetic mismatch distributions were also analyzed to estimate the relative time of population expansion from a simulated sudden-expansion model (Schneider & Excoffier, 1999). This approach is based on the frequency distribution of the number of genetic differences between paired individuals in a population, which follow a unimodal distribution soon after demographic expansion (Rogers & Herpending, 1992). Goodness-of-fit of the mismatch distribution to the simulated sudden-expansion model was examined by *χ*^2^ test, and the number of generations since the population started expanding (*t*) was estimated from *t* = *τ*∕2*u* , where *τ* is an estimate obtained from the mismatch analysis and *u* is the total mutation rate per generation per gene (Schneider & Excoffier, 1999). In addition, Tajima’s *D*, which can be used as an indicator of historical population expansion events, was calculated.

Bayesian Coalescent Skyline Plots were reconstructed by the software BEAST 2.5.0 (Bouckaert et al., 2014). Number of Markoc chain Monte Carlo (MCMC) were changed from 10^7^ to 10^8^ after 10^6^ to 10^7^ burn-in processes. The results were visualized using Tracer v. 1.7.1 (Rambaut, Suchard & Drummond, 2013).

## Results

**Figure 2:**
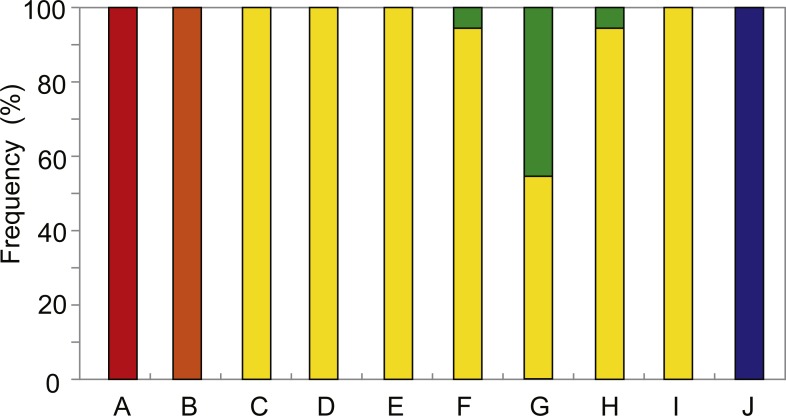
Distribution and local relative abundance of *P. glabra* (red), *P. lucida* (orange), *P. subglabra* (yellow), *P. clathrata* (green), and *P. kuroshimensis* (blue). IDs in the capitalized letters corresponds to location codes in Table 1.

The distribution and relative abundance of the five *Provanna* species inhabiting the surveyed areas were summarized in Fig. 2. Three species were only found in one locality: *P. glabra* in Off Hatsushimas seep site of Sagami Bay, *P. lucida* in Minami-Ensei Knoll, and *P. kuroshimensis* in Kuroshima Knoll (Table 2). *Provanna subglabra* was found in all vent sites except the Minami-Ensei Knoll and *P*. *clathrata* was found in three vent sites (Hakurei site of Izena Hole, Irabu Knoll, and Hatoma Knoll). *Provanna subglabra* was often the only species present and highly dominant when *P. clathrata* co-occurred (Fig. 2); the relative abundance of *P*. *clathrata* was highest in the Irabu Knoll, the deepest of all sites surveyed in the present study.

**Table 2:** Genetic diversity indices of local *Provanna* populations.

ID	Population	*N*	*N*_*h*_	*N*_*p*_	*Ĥ*	*π*	Tajima’s *D*
A	*P. glabra* (Sagami Bay)	22	22	49	1.0000 ± 0.0137	0.006759 ± 0.003681	−1.87288^**^
B	*P. lucida* (Minami-Ensei Knoll)	20	7	18	0.8053 ± 0.0564	0.004194 ± 0.002415	−0.51514
	*P. subglabra*						
C	Iheya North	20	19	56	0.9947 ± 0.0178	0.008499 ± 0.004568	−1.78983^*^
D	Iheya Ridge	16	16	47	1.0000 ± 0.0221	0.009295 ± 0.005036	−1.35916
E	Jade site, Izena Hole	19	16	52	0.9852 ± 0.0223	0.008394 ± 0.004529	−1.69953^*^
F	Hakurei site, Izena Hole	17	16	57	0.9926 ± 0.0230	0.010619 ± 0.005682	−1.46468
G	Irabu Knoll	21	20	53	0.9952 ± 0.0165	0.009180 ± 0.004894	−1.41920
I	Hatoma Knoll	14	14	48	1.0000 ± 0.0270	0.008728 ± 0.004798	−1.75860^*^
J	Dai-yon Yonaguni Knoll	19	18	48	0.9942 ± 0.0193	0.008484 ± 0.004574	−1.46415
	*P. clathrata*						
F	Hakurei site, Izena Hole	1	1	ND	ND	ND	ND
H	Irabu Knoll	17	15	37	0.9853 ± 0.0252	0.005980 ± 0.00343	−1.76989^*^
I	Hatoma Knoll	1	1	ND	ND	ND	ND
K	*P. kuroshimensis* (Kuroshima Knoll)	20	15	19	0.9579 ± 0.0328	0.004129 ± 0.002382	−0.73793

**Notes.**

*N* number of individuals *N*_*h*_ number of haplotypes *N*_*p*_ number of polymorphic sites H haplotype diversity with standard errors *π* nucleotide diversity with standard errors ND not detected

* *P*  <  0.05.

** *P*  < 0.01.

**Figure 3:**
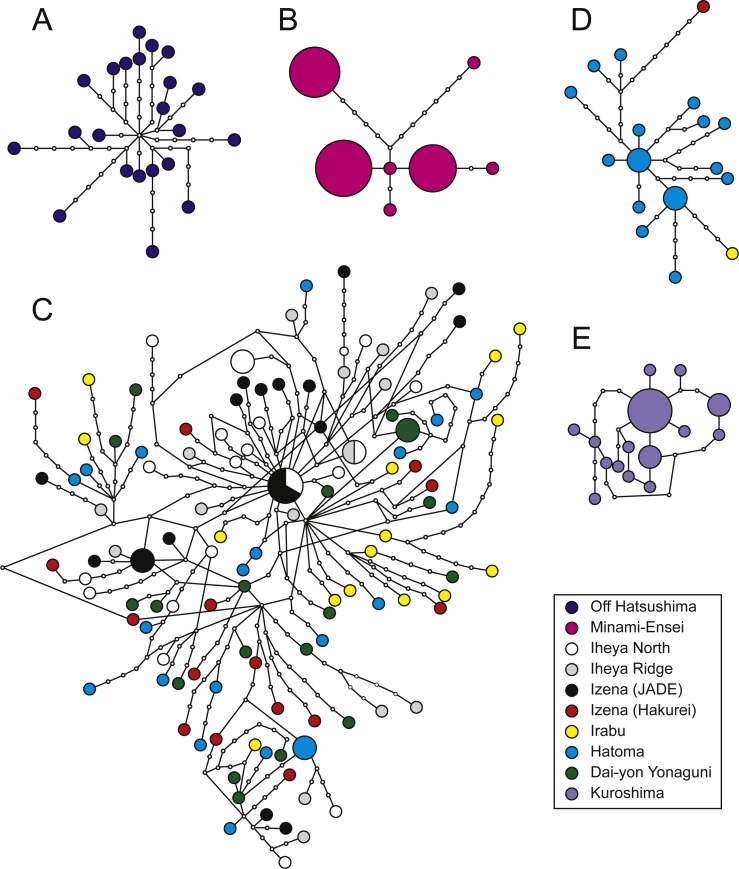
Haplotype networks. *P. glabra* (A), *P. lucida* (B), *P. subglabra* (C), *P. clathratas* (D), and *P. kuroshimensis* (E).

Genetic diversities in local populations were higher in all populations of *P*. *subglabra* among the five species, as inferred from haplotype ($\hat {H}$) and nucleotide (*π*) diversities (Table  2). Simple parsimonious haplotype networks consisting of a few dominant haplotypes were recovered in *P. lucida* and *P. kuroshimensis*, simple networks consisting of diversified haplotypes were recovered in *P. glabra* and *P. clathrata*, while a complicated haplotype network was recovered for *P. subglabra* (Fig. 3). Shared haplotypes were found between local populations of *P. subglabra* in Iheya North field and JADE site in Izena Hole as well as between Iheya North field and Iheya Ridge, but not in populations inhabiting Hakurei site, Hatoma, and Dai-yon Yonaguni Knolls (Fig. 3C). No statistically significant genetic differences were found among the local populations in *P. subglabra* from Φ_*ST*_ and Wright’s exact test (Table 3). The degree of genetic differentiation among local populations was examined only for *P. subglabra*, as that was the only species with sufficient individuals from more than one site (Fig. 2). Pairwise Φ_*ST*_ did not indicate clear genetic structures among local populations (Table 3), and homogeneity in the haplotype distribution was not rejected by Wright’s exact test (*P* > 0.05). However, the result of AMOVA, when assuming a depth subdivision of populations, was statistically significant (*P* < 0.05, among groups variation: 0.18, among populations within groups variation: 0.44, and within populations variation: 99.38), whereas the analyses assuming a latitudinal subdivision did not yield statsiticaly significant differences (*P* > 0.05, among groups variation: 0.08, among populations within groups variation: 0.49, and within populations variation: 99.43).

**Table 3:** Population segregation (pairwise Φ_*ST*_) of *P. subglabra*.

	C	D	E	F	G	I	J
C		–	–	–	–	–	–
D	−0.00046		–	–	–	–	–
E	0.00616	0.00559		–	–	–	–
F	0.00630	0.00369	0.01248		–	–	–
G	0.00264	0.00242	0.01112	0.00604		–	–
I	0.00269	0.00000	0.00895	0.00373	0.00244		–
J	0.00555	0.00295	0.01170	0.00660	0.00530	0.00298	

**Notes.**

*P* > 0.05 for all cells.

Tajima’s *D* was negative in value for all examined populations (Table 2). Among them, the lowest value was observed in *P. glabra* from the Off Hatsushima seep (−1.87288), and the highest value was observed in *P. lucida* from the Minami-Ensei Knoll vent field (−0.51514). The population of *P. kuroshimensis* from the Kuroshima Knoll exhibited a similar Tajima’s *D* value (−0.73793) to that of *P. lucida*, while those of *P. subglabra* and *P.  clathrata* showed values similar (ranged from −1.78983 to −1.35916) to that of *P. glabra*. Values obtained for populations of *P. glabra, P. subglabra* and *P. clathrata* were statistically significant.

**Figure 4:**
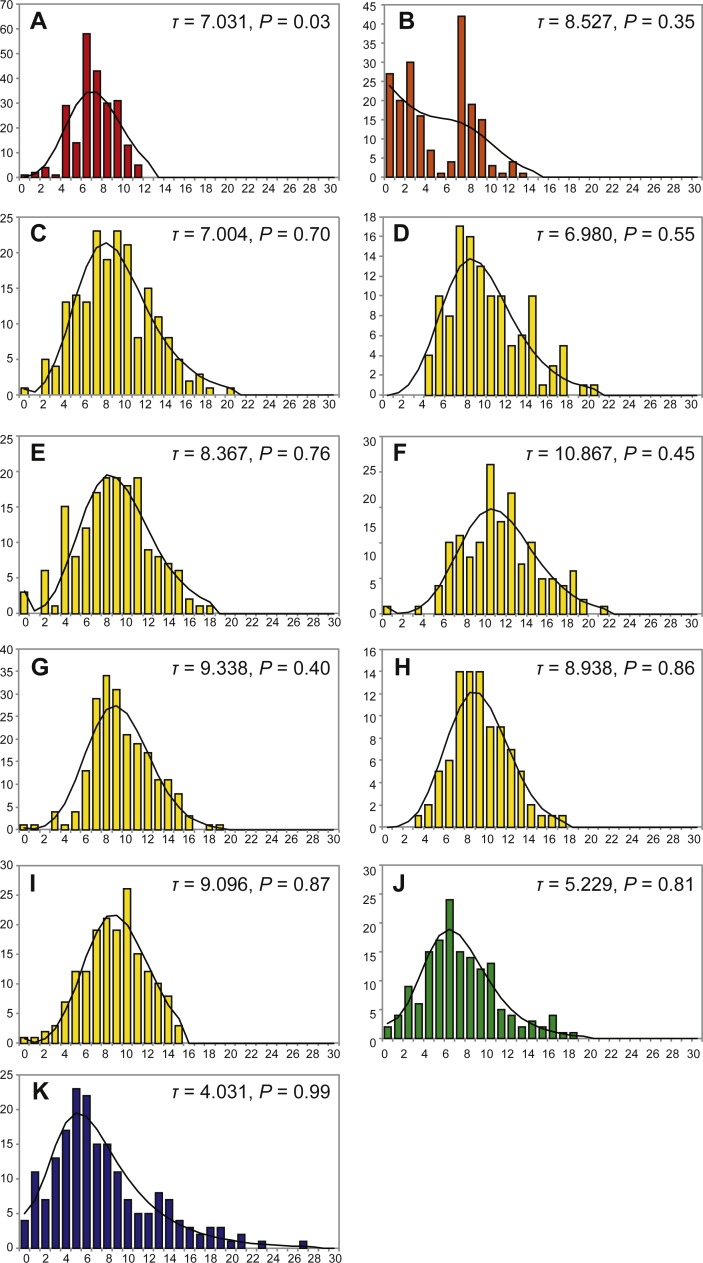
Mismatch distributions of local populations of *Provanna* gastropods, with number of mismatches plotted on horizontal axes and frequency plotted on vertical axes. IDs in the capitalized letters are correlated with those in Table 1. (A) *P. glabra* from Off Hatsushima, (B) *P. lucida* from Minami-Ensei Knoll, (C) *P. subglabra* from Iheya North, (D) *P. subglabra* from Iheya Ridge, (E) *P. subglabra* from Jade site in Izena Hole, (F) *P. subglabra* from Hakurei site in Izena Hole, (G) *P. subglabra* from Irabu Knoll, (H) *P. clathrata* from Irabu Knoll, (I) *P. subglabra* from Hatoma Knoll, (J) *P. subglabra* from Dai-yon Yonaguni Knoll, (K) *P. kuroshimensis* from Kuroshima Knoll.

Mismatch distribution was not statistically significantly different from the expansion model distribution (*P* <  0.05 by *χ*^2^ test), which implies that population expansion occurred in the detectable past, except for a single populations of *P. glabra* from the Off Hatsushima site (Fig. 4). Tau (*τ*) value, an indicator for the relative age of population expansion among closely related taxa, was 7.031 for the *P. glabra* population from the Off Hatsushima seep in Sagami Bay, 8.257 for the *P. lucida* population from Minami-Ensei Knoll, 4.021 for the *P. kuroshimensis* population from Kuroshima Knoll, 5.229 for the *P. clathrata* population from Irabu Knoll, and ranged between 6.980 (Iheya Ridge) and 10.867 (Hakurei site, Izena Hole) for *P. subglabra* (Fig. 4). The mismatch distribution of *P. lucida* in Miami-Ensei Knoll visually exhibited two prominent peaks, but the distribution was not statistically different from the model distribution.

**Figure 5:**
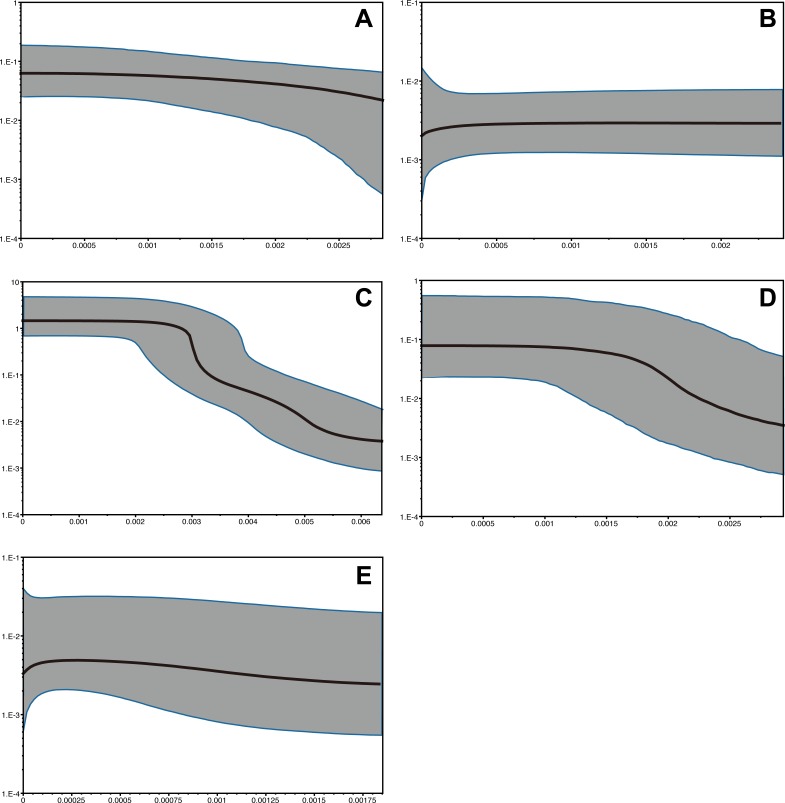
Bayesian skyline plots of *Provanna* gastropods, with time or mutations per sites plotted on the horizontal axis and generation-scaled effective population size on the vertical axis. Black lines indicate median estimates, and shaded zones surrounded by blue lines indicate 95% highest posterior density limits. (A) *P. glabra*, (B) *P. lucida*, (C) *P. subglabra*, (D) *P. clathrata*, (E) *P. kuroshimensis*.

The time range of Bayesian coalescent skyline plot estimated for each species was different, due to the dfferences in the divergence of the sequence dataset available, i.e., the longest in *P. subglabra* with the highest divergence observed, compared to the other species (Fig. 5). Historical demographic increase was inferred for *P. clathrata* and *P. subglabra*, whereas relatively constant demographic histories were inferred for *P. glabra*, *P. lucida*, and *P. kuroshimensis*.

## Discussion

Results from the present study elucidated the distribution and genetic diversity of five *Provanna* species inhabiting chemosynthetic environments in the western Pacific, and revealed the population history and genetic connectivity of their local populations. All five species were restricted to either vent or seep habitats, with three (*P. glabra, P. lucida, P. kuroshimensis*) currently only known from a single site, and the other two (*P. subglabra* and *P. clathrata*) being found in at least three hydrothermal vent fields in the Okinawa Trough (Fig. 2).

Interestingly, both nucleotide diversity (Table 2) and number of mismatches (Fig. 4) were higher in *P*. *subglabra* than in *P. clathrata* at the Irabu Knoll, suggesting that population is more diversified in *P. subglabra* than *P. clathrata*, under the same environment. Both species appear to have experienced sudden expansions of population in the detectable past, according to Tajima’s *D* and mismatch analyses. Furthermore, *τ* in mismatch distributions was higher in *P. subglabra* (*τ* = 8.938) than that in *P. clathrata* (*τ* = 5.229; Figs. 4G and 4H), indicating that the historical demographic increase began earlier in *P. subglabra* than in *P. clathrata*, as shown by the Bysian skyline plot (Fig. 5). One explanation for these results, assuming that the nucleotide substitution rate or life-history traits are comparable in both species, is that *P*. *subglabra* colonized the Okinawa Trough earlier than *P. clathrata*. This interpretation implies that the suitable habitat for *P*. *subglabra* was formed earlier than that for *P. clathrata* during the geological history of the Okinawa Trough. *Provanna clathrata* appears to have adapted to deeper depths and only occurs below 1400 m deep, whereas *P. subglabra* appears to occur throughout a wide depth range (Table 1; also see Miyazaki et al., 2017). This agrees with the fact that depth of the back-arc basin spreading centre increases with time, meaning the deeper sites suitable for *P. clathrata* came into existence later than the shallower sites suitable for *P. subglabra.* This interpretation therefore also highlights the significance of habitat formation during the invasion and colonization process of *Provanna* gastropods. Alternatively, since nucleotide substitution is correlated to the number of offspring produced in a generation and the number of reproductive opportunities per year (e.g., periodic vs. continuous reproduction), the nucleotide substitution rate may not be uniform among *Provanna* species, in which case we cannot exclude that the colonization time of the both species might be the same.

Of the five species surveyed, *P. subglabra* had by far the widest distribution range, being found in all currently examined hydrothermal vent fields in the Okinawa Trough except for the very shallow Minami-Ensei Knoll. At this point, no *Provanna* species have been observed in Yoron Hole, the shallowest hydrothermal field of the Okinawa Trough (550–600 m deep;  Watanabe & Kojima, 2015). Notably, *P*. *subglabra* appears to be segregated into two regional populations, with the northern population inhabiting Iheya North, Iheya Ridge, and Jade site in Izena Hole, while the southern population inhabits Hakurei site in Izena Hole, Irabu Knoll, Hatoma Knoll, and Dai-Yon Yonaguni Knoll. This segregation can be seen in pairwise Φ_*ST*_ (Table 3) and parsimonious haplotype networks (Fig. 4), although not on the results of Wright’s exact tests.

Though this genetic structure of *P. subglabra* initially seems to be a northern-southern segregation, the two populations are separated within the Izena Hole vent field where Jade and Hakurei sites are within 3 km of each other in the single caldera (Kawagucci et al., 2010; Ishibashi et al., 2014). Genetic structure across such a close distance has not been observed in other hydrothermal vent animals in the Okinawa Trough examined to date (e.g., the gastropod *Lepetodrilus nux* (Nakamura et al., 2014), *Neoverruca* barnacles (Watanabe et al., 2005), alvinocaridid shrimps (Yahagi et al., 2015). Typically, these genetic segregations are seen in larger scales in other Pacific chemosynthetic ecosystems, such as inter-current microplates, fracture zones, and topological depressions in the eastern Pacific (Won et al., 2003; Hurtado, Lutz & Vrijenhoek, 2004; Johnson et al., 2006; Plouviez et al., 2009; Vrijenhoek, 2010), or between backarc basins in the western Pacific ( Kojima et al., 2000;  Kojima et al., 2001; Watanabe et al., 2005). Therefore, the segregation of *P*. *subglabra* on either side of the Izena Hole is unlikely to be caused by distance. There was also no significant differences between northern and southern populations of *P. subglabra* when compared using AMOVA.

Instead, genetic segregation seen in *P. subglabra* is likely caused by environmental differences, particularly depth. This is supported by a significant difference in the AMOVA analysis between shallower and deeper populations. There is a clear gap in water depth between Jade (1,309 m) and Hakurei (1,617 m) sites in the Izena Hole. Furthermore, the distribution of Okinawa Trough *Provanna* species seems to be separated by depth, with *P. lucida* in the shallowest Minami-Ensei Knoll, *P. subglabra* in intermediate depths, and *P.  clathrata* with the deepest central bathymetric range. Though chemistry of hydrothermal fluids, such as concentration of gases, cannot be excluded entirely as a possibility, this is unlikely to be the key environmental difference causing the segregation in *P. subglabra.* In the Okinawa Trough, subseafloor phase separation leads to intra-field diversity of mineralization which can cause drastic differences in hydrothermal fluid chemistry in different sites (Ishibashi et al., 2015). However, in Izena Hole, the geochemistry of high-temperature vent fluids is generally similar between Jade and Hakurei sites, except for the concentration of minor gases including hydrogen (Ishibashi et al., 2014). This difference in hydrogen, however, is caused by whether the rising geofluid passes through a sediment layer or not, and not related to depth or latitude (Ishibashi et al., 2014; Kawagucci, 2015).

Genetic diversity and mismatch distribution were relatively low for *P. lucida* from Minami-Ensei Knoll and *P. kuroshimensis* from Kuroshima Knoll, and statistical analyses did not indicate recent population expansion, with Tajima’s *D* being statistically insignificant for both species, and constant demographic states shown by Bayesian skyline plots. These results together suggest that these populations are fragmented or consist of relatively small populations compared to those observed in the other species. The distribution of these two species is restricted to depths shallower than 1,000 m, shallower than the other *Provanna* species examined. Planktonic larval duration, which is important in mediating metapopulation, is not known for *Provanna* gastropods but has been shown for 69 marine organisms to have a trade-off relationship with temperature with the mean dispersal distance increasing from 20 to 225 km as the temperature drops from 30 to 5 °C (O’Connor et al., 2007). Therefore, species in shallower waters tend to have shorter planktonic larval durations, and thus shorter dispersal distances, especially in patchy habitats like hydrothermal vent fields and hydrocarbon seep sites. On the other hand, the population of *P. glabra* in the Off Hatsushima seep site exhibited comparable genetic diversities to those in *P. subglabra*, with a clear population expansion in the past (Tajima’s *D* in Table 2 and mismatch distribution in Fig. 4A).

Bayesian skyline plots (Fig. 5) inferred differences in demographic history between the comparatively deeper-occuring species (*P. clathrata* and *P. subglabra* with historical demographic increase) and shallower-occuring species (*P. glabra*, *P. lucida*, and *P. kuroshimensis* with constant demographic histories). There was no clear pattern that matched habitat types (i.e., seep vs. vent), further implying that depth is a key factor in shaping the past population dynamics of *Provanna* species instead of habitat types (i.e., vents vs. seeps).

The phylogenetic analysis of *Provanna* based on partial DNA sequences of COI, 16SrRNA, and 28SrRNA showed that *P. glabra* could not be separated from *P. laevis* in Monterey Bay (Sasaki et al., 2016). This suggests that the population of *P. glabra* in the Off Hatsushima site in fact is part of a large and complex metapopulation across the Pacific Ocean (*P. laevis* in Monterey Bay being the other end), with comparable genetic diversity to *P. subglabra*. More detailed morphological and genetic analyses, particularly samples from intervening populations, are required to solve this problem.

## Conclusion

Genetic analyses of partial mitochondrial COI sequences revealed differences in the historical timing of colonization among *Provanna* species inhabiting vents and seeps in the northwestern Pacific. Our results indicate that depth is likely the key factor in initial colonization, niche separation, distribution determination, and speciation for these gastropods. Additional characterization of life-history traits (such as dispersal depth and larval duration; see Yahagi et al., 2017) is, however, required for confirming our hypotheses and further advance our understanding of how biodiversity is generated in deep chemosynthetic ecosystems.

##  Supplemental Information

10.7717/peerj.5673/supp-1 Table S1 List of DNA sequences obtained in the present study

## Additional Information and Declarations

### Field Study Permissions

The following information was supplied relating to field study approvals (i.e., approving body and any reference numbers): The samples were collected by JAMSTEC, which is a Japanese government research institution, in Japanese waters (Exclusive Economic Zone). We routinely submit collecting permit requests to the relevant prefectural governments, in this case Okinawa Prefecture and Kanagawa Prefecture. As both prefectures replied that no permits are required, no permits were needed for the collection.

### DNA Deposition

The following information was supplied regarding the deposition of DNA sequences: The COI sequences are available at GenBank with accession numbers AB810040 to AB810216.
